# Systematic review of venous thromboembolism (VTE) occurrence in hospitalized patients receiving prophylactic unfractionated heparin twice vs. three times daily

**DOI:** 10.1007/s11239-025-03137-8

**Published:** 2025-06-26

**Authors:** Stephanie H. Flint, Ashley E. Woodruff, Molly K. Maloney, Maya R. Chilbert

**Affiliations:** 1 School of Pharmacy and Pharmaceutical Sciences, University at Buffalo, Buffalo, NY USA; 2 Buffalo General Medical Center, Buffalo, NY USA; 3 University Libraries, University at Buffalo, Buffalo, NY USA

**Keywords:** Unfractionated heparin, Prophylaxis, Venous thromboembolism, Dosing frequency, Hospitalized patients

## Abstract

Guidelines recommend 5000U subcutaneous unfractionated heparin (UFH) for venous thromboembolism (VTE) prophylaxis in acutely ill hospitalized adults, but data comparing dosing frequencies is limited. This systematic review aimed to compare VTE and bleeding outcomes between twice daily (BID) and three times daily (TID) UFH regimens. A literature search was completed on 3/7/2024. The primary outcome was VTE occurrence (deep vein thrombosis (DVT) or pulmonary embolism (PE)). Secondary outcomes included bleeding events. Studies reporting any relevant outcomes were included, while non-human studies, reviews, non-English texts, and high VTE risk populations were excluded. Risk of bias was assessed using the Cochrane Risk-of-Bias or Newcastle-Ottawa Quality Assessment Form. Data were synthesized using Covidence and Excel. After screening, 24 studies were included: 9 observational and 15 randomized studies. Regimens with TID UFH had a 3.1% VTE occurrence (12 studies, *n* = 145/4653) compared to 4.0% with BID regimens (9 studies, *n* = 218/5426). Three times daily regimens demonstrated 4.8% DVTs (11 studies, *n* = 244/5102) and 0.4% PEs (11 studies, *n* = 24/5372), compared to 9.7% DVTs (11 studies, *n* = 199/2062) and 0.9% PEs (9 studies, *n* = 17/1974) with BID regimens. Bleeding events occurred in 3.2% of patients with BID (9 studies, *n* = 196/6080) and 4.3% with TID regimens (13 studies, *n* = 393/9044). Three times daily UFH regimens led to fewer VTE, DVT, and PE events but more bleeding compared to BID. Newer data suggests BID dosing may be more appropriate for general medical populations. Limitations include variability in data quality and publication dates. Registered with PROSPERO. No funding was received.

## Key points

• Current guidelines recommend no preference between BID or TID 5000U SC UFH for VTE prophylaxis in acutely ill hospitalized adult patients on medical floors.

• In this systematic review, patients on TID regimens experienced fewer VTEs (3.1%) but more bleeds (4.3%) than patients on BID regimens (4.0% VTEs, 3.2% any bleed).

• In the subgroup analysis of higher quality studies, contemporary good quality observational data showed similar and lower occurrence of VTE between regimens (1.7% TID, 1.2% BID), with sustained differences in any bleeding occurrence.

• In the majority of acutely ill hospitalized adults, BID regimens may be appropriate to balance thrombotic and bleeding risk. However, the increased thrombotic burden in patients admitted to medical floors with acute MI or stroke may warrant TID dosing.

• This review highlights the importance of the publication of newer observational studies comparing VTE prophylactic regimens to assess patient outcomes of VTE and bleeding in current practice.

## Graphical abstract

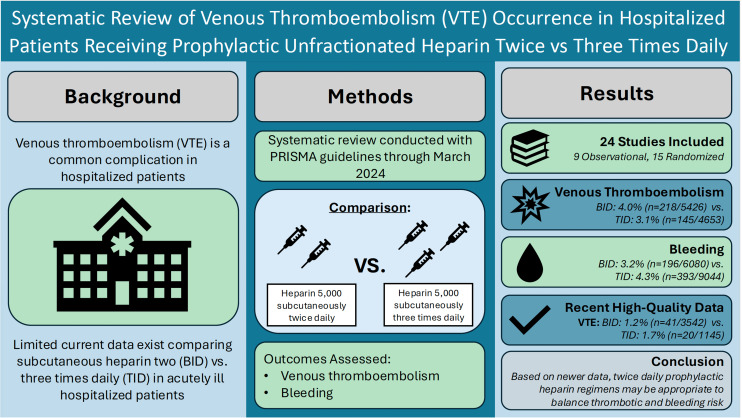

## Introduction

 Venous thromboembolism (VTE), including both deep vein thrombosis (DVT) and pulmonary embolism (PE), is a common complication in hospitalized patients [1]. An estimated 375,000 to 425,000 new cases of VTE occur every year in the United States, which is associated with an approximate cost of $7–10 billion [2]. Acutely ill hospitalized patients are at an increased risk of VTE development due to reduced mobilization in the hospital setting combined with acute illness that impacts their risk of thrombosis [3]. Therefore, guidelines recommend the use of pharmacologic VTE prophylaxis in most acutely ill medical patients to prevent such events [4, 5].

The American Society of Hematology and American College of Chest Physicians suggest using pharmacologic prophylaxis over mechanical prophylaxis for VTE prevention in acutely ill medical patients [4, 5]. Multiple agents can be used, and unfractionated heparin (UFH) is generally preferred for patients with kidney impairment or pending procedures [6]. Despite the continued utilization of low-dose subcutaneous (SC) UFH, guidelines recommend the use of twice daily (BID) or three times daily (TID) dosing without a preference for one dose over the other due to limited data comparing these regimens [4, 5].

The most recent review of the literature comparing SC BID and TID prophylactic UFH dosing was a meta-analysis performed by Phung et al. in 2011 [7]. Since this time, multiple observational studies comparing VTE prophylactic dosing regimens have been published, warranting a re-evaluation and synthesis of the literature. The objective of this study is to summarize evidence on VTE and bleeding outcomes in acutely ill hospitalized adult patients receiving BID or TID 5000U SC prophylactic UFH.

## Methods

This systematic review adhered to the Preferred Reporting Items for Systematic Review and Meta-Analysis (PRISMA) statement and was registered with PROSPERO (CRD42023493327) [8].

### Search strategy

In line with the recommendations in the Cochrane Handbook for Systematic Reviews, the following databases and registries were searched: PubMed, Embase (Elsevier), CINAHL Plus (EBSCOhost), Web of Science Core Collection, the Cochrane Central Register of Controlled Trials (OVID EBM Reviews), and ClinicalTrials.gov [9]. Databases were selected to optimize coverage of relevant research literature. Searches were run on 7 March 2024 and were limited to English language with no publication date restrictions. The searches were developed and performed by a health sciences librarian (M. K. M.) using a combination of controlled vocabulary and keywords on subcutaneous heparin, two- or three-times daily dosing, and venous thromboembolism. Terms were identified through crosschecking preliminary articles which met the inclusion criteria with controlled vocabularies (e.g., Medical Subject Headings in PubMed and Emtree in Embase) to build a comprehensive search. In platforms without controlled vocabulary, only keywords were used. Full search strategies are shared via Open Science Framework.

### Inclusion and exclusion criteria

Studies were included in the review if patients were acutely ill hospitalized adults (≥ 18 years old) on medical floors who required VTE prophylaxis. Prophylactic regimens of SC UFH 5000U TID and/or SC UFH 5000U BID were required for inclusion. Studies were excluded if patients were < 18 years old; pregnant; admitted to a surgery, critical care, or trauma floor; had COVID-19, malignancy, extremes of weight (< 18 kg/m^2^ body mass index (BMI) or > 40 kg/m^2^ BMI), spinal cord injury, burns, or bleed at time of VTE prophylaxis initiation. Case reviews, case series, review articles, commentaries, editorials, and letters were excluded, but their reference lists were examined to identify potentially relevant studies. Articles in languages other than English, non-human studies, and unpublished data were also excluded.

The primary outcome of interest was occurrence of any VTE during the inpatient stay while receiving SC UFH prophylactic therapy, where VTE included a diagnosis of either DVT or PE. Secondary outcomes assessed safety through the occurrence of bleeding. Any study that reported bleeding during the inpatient stay while receiving SC UFH prophylactic therapy was included in the safety outcome of any bleeding. Where possible, events were classified in accordance with the International Society on Thrombosis and Hemostasis (ISTH) criteria for major bleeding in non-surgical patients [10].

### Study selection, data extraction, and quality assessment

Two independent researchers (M. R. C. and S. H. F.) screened all titles and abstracts with disagreements resolved by a third author (A. E. W.) using Covidence [11]. Full text was then retrieved. For clinical trial registrations and conference abstracts included, searches were conducted to locate available published data. The same screening procedure described above was used to review the full text, and reasons for exclusion were recorded. Unpublished data, full text articles not available in English, and duplicate reports of trials identified during full text review were excluded.

As with study selection, two independent researchers (M. R. C. and S. H. F.) extracted information and performed a quality assessment on all included articles using a template built in Covidence. The template included study characteristics (first author, publication year, trial design, UFH dosing regimen, patient population, and VTE screening approach), patient characteristics (number of participants, age, sex, weight, ethnicity/race, and treatment period), primary outcomes (VTE occurrence, DVT occurrence, and PE occurrence), secondary outcomes (bleeding occurrence and bleeding definition), and quality assessment. In cases of missing data, no data were extracted for synthesis, and the total n for each datapoint was determined with available data. Patient populations were classified for each study as either a general medical population, neurologic for ischemic stroke admissions, or cardiac for myocardial infarction (MI) admissions. The VTE screening approach used in each study was classified as serial if researchers screened for VTE events on a consistent basis in all patients, or symptom-based if researchers only screened for VTE events upon patient presentation of symptoms. If researchers used a mixture of these approaches, both serial and symptom-based were recorded. Study quality and risk of bias was assessed using either the Newcastle-Ottawa Quality Assessment Form for Cohort Studies or the Cochrane Risk-of-Bias Tool for Randomized Trials [12, 13]. The Newcastle-Ottawa Form classified observational studies as either good quality, fair quality, or poor quality [12]. The Cochrane Risk-of-Bias Tool classified randomized studies as either low risk of bias, some concerns of bias, or high risk of bias [13]. Two researchers (M. R. C. and S. H. F.) reviewed extraction for consensus and to resolve any conflicts. Extracted qualitative data and quality assessments were exported to Excel and summarized in tables.

### Synthesis of results

Quantitative results for the primary and secondary outcomes were synthesized in Excel. Studies were eligible for outcome synthesis if they reported the occurrence of an event and the patient population from which it was collected. When possible, intention-to-treat (ITT) or per-protocol populations were used for VTE outcomes, and total populations or safety populations were used for bleeding outcomes. For studies that reported both ITT and per-protocol populations for VTE outcomes, per-protocol populations were used. If a study reported both suspected and confirmed VTE events, confirmed events were used. For the primary outcome, if a total VTE occurrence was not reported, reviewers combined the DVT and PE events as long as they were measured in the same population to calculate total VTE. For the secondary outcome of any bleeding, the same approach was taken to add all reported bleeding events from the same population. For the secondary outcome of ISTH major bleeding, study definitions of bleeding were assessed against the ISTH major bleeding definition. If a study’s definition of bleeding included the ISTH major bleeding definition, only the bleeding events that met the ISTH definition were included in the synthesis for this outcome. Quality of studies included in each synthesized outcome were reported according to the results of individual quality assessments.

### Subgroup analysis

Three subgroup analyses of the data were performed to further analyze results. The general medical population subgroup analysis only included patients admitted to a general medicine floor without a primary cardiac or neurologic diagnosis of MI or ischemic stroke. This allowed analysis with and without cardiac/neurologic subgroups as they have additional thromboembolic risk. The higher data quality subgroup analysis only included randomized studies that were rated as low or some concerns of bias, and observational studies that were rated as good or fair quality. High risk of bias and poor-quality studies were excluded to assess differences in results that may be attributed to a large proportion of lower quality data. Lastly, a subgroup analysis assessing contemporary observational data was conducted to best reflect current practice and highlight data published after the prior systematic review.

## Results

### Search results

**Fig. 1:**
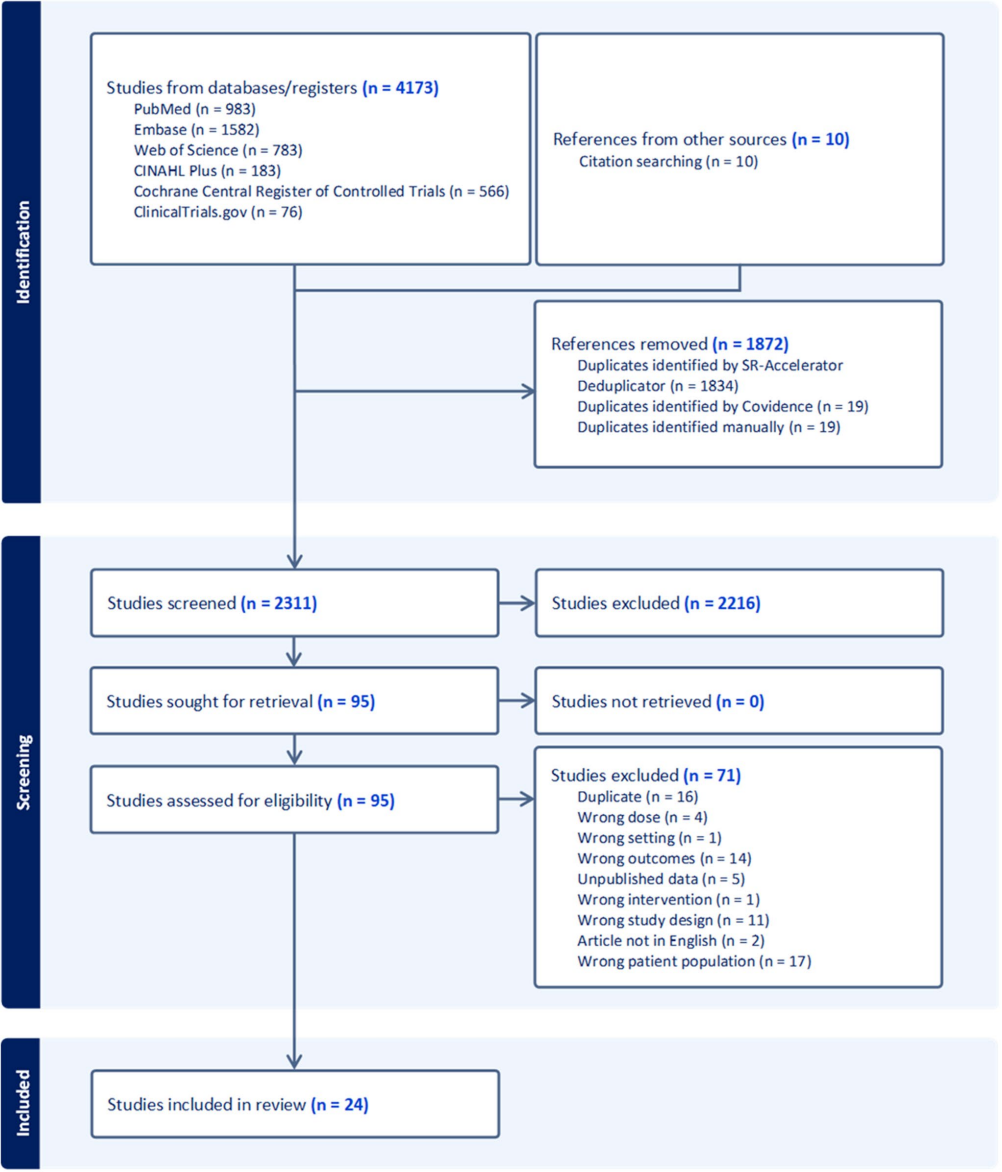
PRISMA flow chart

Search and screening results are shown in the PRISMA diagram (Fig. 1). Searching returned 4173 results with 1872 removed as duplicates using Systematic Review Accelerator’s Deduplicator and Covidence [11, 14]. Citation searching identified an additional ten articles, resulting in a total of 2311 items. Screening resulted in the exclusion of 2216 studies, and full text was retrieved for the remaining 95 studies. An additional 71 studies were excluded during full text review primarily due to incorrect patient population (*n* = 17), duplicate (*n* = 16), and wrong outcomes (*n* = 14), leaving 24 studies eligible for inclusion [15–38].

### Study characteristics

Of the 24 included studies (*n* = 16101), there were 9 observational studies and 15 randomized studies spanning publication years of 1973 to 2023 (Table 1). Ten studies included a BID 5000U SC UFH regimen, 11 studies included a TID 5000U SC UFH regimen, and 3 studies included both BID and TID regimens. The VTE screening approach was serial in 12 studies, symptom-based in 8 studies, both serial and symptom-based in 3 studies, and not reported in 1 study. Of the 9 observational studies, 4 (44.4%) were rated good quality, 0 (0.0%) were rated fair quality, and 5 (55.6%) were rated poor quality by the Newcastle-Ottawa Quality Assessment Form. Of the 15 randomized studies, 4 (26.7%) were rated low risk of bias, 5 (33.3%) were rated some concerns of bias, and 6 (40.0%) were rated high risk of bias by the Cochrane Risk-of-Bias Tool. Additional baseline characteristics of the patients included in each study are reported in Table 2.

**Table 1:** Study characteristics

Last Name (Publication Year)	Study Design	Included 5000U SC UFH Dosing Regimens	Patient Population	VTE Screening Approach	Quality of Study^a^
*Observational Studies*					
Kamran (1998) [15]	Prospective, interventional, non-randomized	BID	Neurologic	Symptom-based	Poor quality
Peterman (2006) [16]	Prospective observational	BID	General Medical	Symptom-based	Poor quality
Ishida (2014) [17]	Retrospective cohort	BID	General Medical	Serial	Poor quality
Beall (2016) [18]	Retrospective cohort	TID	General Medical	Symptom-based	Poor quality
Joy (2016) [19]	Single-center, retrospective, observational cohort	TID	General Medical	Symptom-based	Good quality
Regis (2021) [20]	Retrospective cohort	TID	General Medical	Symptom-based	Good quality
Sorgi (2022) [21]	Retrospective cohort	BID, TID	General Medical	Symptom-based	Good quality
Jatis (2023) [22]	Single-center, retrospective, nested case-control	BID, TID	General Medical	NR	Good quality
Kaylor (2023) [23]	Retrospective cohort	BID, TID	General Medical	Symptom-based	Poor quality
*Randomized Studies*					
Warlow (1973) [24]	Double-blind randomized	BID	Cardiac	Serial	Some concerns of bias
Gelmers (1980) [25]	Unblinded randomized controlled	BID	Neurologic	Symptom-based	High risk of bias
Belch (1981) [26]	Randomized controlled	TID	General Medical	Serial	High risk of bias
McCarthy (1986) [27]	Prospective randomized	TID	Neurologic	Serial	High risk of bias
Zawilska (1989) [28]	Prospective randomized	BID	Cardiac	Serial	High risk of bias
Harenberg (1990) [29]	Double-blind, randomized, controlled	TID	General Medical	Serial	Some concerns of bias
Turpie (1992) [30]	Double-blind randomized	BID	Neurologic	Serial	High risk of bias
Dumas (1994) [31]	Prospective, double-blind, randomized	BID	Neurologic	Serial	Low risk of bias
Bergmann (1996) [32]	Multicenter, double-blind, parallel group, randomized	BID	General Medical	Serial	Low risk of bias
Lechler (1996) [33]	Multicenter, double-blind, randomized controlled	TID	General Medical	Symptom-based, Serial	Some concerns of bias
Hillbom (2002) [34]	Multicenter, double-blind, randomized	TID	Neurologic	Symptom-based, Serial	High risk of bias
Kleber (2003) [35]	Multicenter, open, parallel group, randomized, controlled	TID	General Medical	Serial	Some concerns of bias
Diener (2006) [36]	Multicenter, double-blind, parallel group, randomized, active-controlled	TID	Neurologic	Symptom-based, Serial	Low risk of bias
Sherman (2007) [37]	Open, randomized, controlled	BID	Neurologic	Serial	Some concerns of bias
Riess (2010) [38]	Multicenter, double-blind, randomized, active-controlled	TID	General Medical	Serial	Low risk of bias

^a^observational studies were assessed with the Newcastle-Ottawa Quality Assessment Form for Cohort Studies, randomized studies were assessed with the Cochrane Risk-of-Bias Tool for Randomized Trials

**Table 2:** Study outcomes

Last Name (Publication Year)	*n*	Mean Age (SD), yrs	Male, no. (%)	Mean Weight, kg (SD), or BMI, kg/m^2^ (SD)	Ethnicity/Race, no. (%)	Treatment Period or Length of Stay, days, mean (SD)	VTE, no. (%)	DVT, no. (%)	PE, no. (%)	Any Bleed, no. (%)	ISTH Major Bleeding, no. (%)
**Studies with BID UFH Regimen**											
*Observational Studies*											
Kamran (1998) [15]	681	Group A: 69.3 Group B: 69.5 Group C: 67.6	639 (93.8)	NR	NR	Group A: 14.6 (0.7)^g^ Group B: 14.3 (0.8)^g^ Group C: 14.1 (NR)^g^	30 (4.4)^i^	24 (3.5)	6 (0.9)	NR	NA
Peterman (2006) [16]	92	59.3 (17.9) (*n* = 179)^b^	72 (40.2) (*n* = 179)^b^	Weight: 83.3 (29) BMI: 28.7 (8.8) (*n* = 179)^b^	African-American: 102 (57.0) Caucasian: 73 (40.8) Other: 4 (2.2) (*n* = 179)^b^	7.2 (5.4)^g^ (*n* = 179)^b^	1 (1.1)^i^	1 (1.1)	0 (0.0)	1 (1.1)^j^	NA
Ishida (2014) [17]	93	45.0 (16.4)	54 (58.1)	NR	NR	NR	11 (11.8)^i^	11 (11.8)	0 (0.0)	NR	NA
Sorgi (2022)^a^ [21]	3542	59.3 (17)	1877 (53.0)	Weight: 83.4 (24.4)	NR	6.0 (5.4)^g^	41 (1.2)	NR	NR	105 (3.0)^i^ Major^k^: 25 (0.7) Minor^l^: 80 (2.3)	25 (0.7)
Jatis (2023)^a^ [22]	1151	67 (52–78)^c, d^ (*n* = 3736)^b^	648 (17.3) (*n* = 3736)^b^	Weight: 52.0 (6.3) BMI: 20.4 (3.3) (*n* = 3736)^b^	NR	4.5 (3.1–6.8)^c, d^ (*n* = 3736)^b^	NR	NR	NR	13 (1.1)^m^	NA
Kaylor (2023)^a^ [23]	18	64.7 (16.8) (*n* = 158)^b^	29 (18.4) (*n* = 158)^b^	Weight: 47.1 (6.1) BMI: 19.4 (3) (*n* = 158)^b^	NR	6.8 (3.7) (*n* = 158)^b^	1 (5.6)	0 (0.0)	1 (5.6)	0 (0.0) Major^k^: 0 (0.0) Minor^n^: 0 (0.0)	0 (0.0)
*Randomized Studies*											
Warlow (1973) [24]	63	NR	NR	NR	NR	NR	2 (3.2)^i^	2 (3.2)	0 (0.0)	NR	NA
Gelmers (1980) [25]	52	66.8 (10.4) (*n* = 42)	NR	NR	NR	17.3 (9.2) (*n* = 42)	1 (1.9)^i^	1 (1.9)	0 (0.0)	NR^o^	NA
Zawilska (1989) [28]	50	58 (23–83)^e^	44 (88.0)	NR	NR	NR	NR	2 (4.0)	NR	0 (0.0)^p^	NA
Turpie (1992) [30]	42	72.5 (39–94)^e^	16 (38.1)	NR	White: 41 (97.6) Other: 1 (2.4)	11.9 (1–15)^e^	NR	13 (31.0)	NR	1 (2.4)^i^ Major^k^: 0 (0.0) Minor^q^: 1 (2.4)	0 (0.0)
Dumas (1994) [31]	90	72.9 (13.1)	38 (42.2)	Weight: 66.7 (13.6)	NR	NR	NR	17 (19.8) (*n* = 86)	4 (4.4)	2 (2.2)^i^ Major Hemorrhagic Conversion^p^: 1 (1.1) Minor Hemorrhagic Conversion^p^: 1 (1.1)	NA
Bergmann (1996) [32]	223	82.6 (0.46)^f^	60 (26.9)	Weight: 57.0 (0.78)^f^	NR	9.5 (0.1)	10 (4.6) (*n* = 216)	10 (4.6) (*n* = 216)	0 (0.0) (*n* = 216)	4 (1.8) Major^k^: 2 (0.9) Minor^r^: 2 (0.9)	2 (0.9)
Sherman (2007) [37]	878	66.1 (12.9)	473 (53.9)	BMI: 27.0 (5.3)	White: 523 (59.6) Asian: 193 (22.0) Hispanic: 68 (7.7) Black: 55 (6.3) Other: 39 (4.4)	10.5 (3.2)	121 (18.1) (*n* = 669)	118 (17.6) (*n* = 669)	6 (0.9) (*n* = 669)	70 (8.0) Major Extracranial Hemorrhage^s^: 0 (0.0) Minor Extracranial Hemorrhage^q^: 48 (5.5) Intracranial Hemorrhage: 6 (0.7) (*n* = 872)	NA
**Studies with TID UFH Regimen**											
*Observational Studies*											
Beall (2016) [18]	2182	58 (14.3)	833 (38.2)	NR	NR	4 (1-188)^c, e,g^	5 (0.2)	3 (0.1)	2 (0.1)	2 (0.1) Major^k^: 0 (0.0) Minor^t^: 2 (0.1)	0 (0.0)
Joy (2016) [19]	335	BMI 25-29.9: 55 (12) BMI 30-34.9: 57 (15) BMI 35-39.9: 53 (14)	246 (73.4) BMI 25-29.9: 39 BMI 30-34.9: 103 BMI 35-39.9: 104	Weight: BMI 25-29.9: 102 (11) BMI 30-34.9: 106 (7) BMI 35-39.9: 114 (11)	NR	BMI 25-29.9: 3 (2–7)^c, d,g^ BMI 30-34.9: 4 (2–8)^c, d,g^ BMI 35-39.9: 3 (2–6)^c, d,g^	5 (1.5)	4 (1.2)	1 (0.3)	93 (27.8)^i^ Major^u^: 73 (21.8) Minor^v^: 20 (6.0)	NA
Regis (2021) [20]	164	56 (15)	82 (50.0)	Weight: 110 (23) BMI: 39 (7.7)	Spanish/Hispanic/Latino: 54 (32.9)	7.9 (7.3)^g^	3 (1.8)	NR	NR	47 (28.7)^w^	NA
Sorgi (2022)^a^ [21]	646	61.8 (13.9)	361 (55.9)	Weight: 88.9 (27.3)	NR	5.6 (4.9)^g^	12 (1.9)	NR	NR	17 (2.6)^i^ Major^k^: 5 (0.8) Minor^l^: 12 (1.9)	5 (0.8)
Jatis (2023)^a^ [22]	2574	67 (*n* = 3736)^b^	648 (17.3) (*n* = 3736)^b^	Weight: 52.0 (6.3) BMI: 20.4 (3.3) (*n* = 3736)^b^	NR	4.5 (3.1–6.8)^c, d^ (*n* = 3736)^b^	NR	NR	NR	22 (0.9)^m^	NA
Kaylor (2023)^a^ [23]	140	64.7 (16.8) (*n* = 158)^b^	29 (18.4) (*n* = 158)^b^	Weight: 47.1 (6.1) BMI: 19.4 (3) (*n* = 158)^b^	NR	6.8 (3.7) (*n* = 158)^b^	3 (2.1)	1 (0.7)	2 (1.4)	18 (12.9) Major^k^: 8 (5.7) Minor^n^: 10 (7.1)	8 (5.7)
*Randomized Studies*											
Belch (1981) [26]	50	66.6	35 (70.0)	NR	NR	8^h^	2 (4.0)^i^	2 (4.0)	0 (0.0)	11 (22.0)^i^ Epistaxis: 1 (2.0) Excessive Bruising^x^: 10 (20.0)	NA
McCarthy (1986) [27]	144	76.7 (7.9)	60 (41.7)	NR	NR	NR	39 (27.1)^i^	32 (22.2)	7 (4.9)	4 (2.8)^y^	NA
Harenberg (1990) [29]	82	65.8 (9.9)	35 (42.7)	Weight: 69.3 (12.4)	NR	7-12^e^ (*n* = 166)^b^	0 (0.0) (*n* = 83)	0 (0.0) (*n* = 83)	0 (0.0) (*n* = 83)	NR^o^	NA
Lechler (1996) [33]	482	74 (13)	178 (36.9)	Weight: 66 (15)	NR	NR	5 (1.3) (*n* = 377)	4 (1.1) (*n* = 377)	4 (1.1) (*n* = 377)	15 (3.1) Major^k^: 7 (1.5)	7 (1.5)
Hillbom (2002) [34]	106	69 (10)	59 (55.7)	Weight: 77 (16)	NR	NR	25 (34.7) (*n* = 72)	24 (33.3) (*n* = 72)	3 (4.2) (*n* = 72)	26 (24.5)^i^ Major^k^: 0 (0.0) Minor^t^: 2 (1.9) Injection Site Hematoma^x^: 4 (3.8) Hemorrhagic Conversion: 20 (18.9) Intracerebral Hemorrhage: 0 (0.0)	0 (0.0)
Kleber (2003) [35]	333	70 (14)	183 (55.0)	Weight: 71 (16)	NR	9.6 (2.6)	22 (10.4) (*n* = 212)	21 (9.9) (*n* = 212)	1 (0.5) (*n* = 212)	54 (16.2)^i^ Major^k^: 1 (0.3) Minor^p^: 11 (3.3) Injection Site Hematoma^x^: 42 (12.6)	1 (0.3)
Diener (2006) [36]	273	67.3 (10.6)	164 (60.1)	Weight: 78.4 (13.3) BMI: 27.1 (3.9)	NR	13.6 (3.2)	24 (9.7) (*n* = 248)	23 (9.3) (*n* = 248)	1 (0.4) (*n* = 248)	10 (3.7) Major^k^: 5 (1.8) Minor^t^: 5 (1.8)	5 (1.8)
Riess (2010) [38]	1615	78.7 (6.3)	655 (40.6)	Weight: 71.9 (15.3)	Caucasian: 1597 (98.9)	9.0 (3.3)	NR	130 (10.3) (*n* = 1259)	3 (0.2) (*n* = 1529)	74 (4.6) Major^k^: 10 (0.6) Minor^t^: 65 (4.0)	10 (0.6)

^a^study reported both BID and TID outcomes; ^b^entire study population including other prophylactic regimens; ^c^median; ^d^interquartile range; ^e^range; ^f^standard error of the mean; ^g^length of stay; ^h^duration of scanning; ^i^outcome synthesized by combining DVT and PE rates, or by combining types of bleeding rates; ^j^epistaxis; ^k^ISTH Major Bleeding: fatal bleeding, and/or symptomatic bleeding in a critical area or organ, such as intracranial, intraspinal, intraocular, retroperitoneal, intra-articular or pericardial, or intramuscular with compartment syndrome, and/or bleeding causing a fall in hemoglobin (Hgb) levels of 20 g/L or greater, or leading to a transfusion of 2 U or more of whole blood or packed red blood cells (PRBCs); ^l^clinically-relevant non-major bleed; ^m^decrease in Hgb of 2 g/dL or greater and transfusion of 1 or more units of PRBCs or whole blood within 24 h, or bleeding at critical site (intracranial, intraspinal, intraocular, pericardial, intraarticular, intramuscular with compartment syndrome, retroperitoneal, thoracic, airway bleeding), and have resulted in interruption of UFH for 24 h or more; ^n^clinically-relevant minor bleeding; ^o^not reported in review due to unclear patient population; ^p^not defined; ^q^overt non-major bleed; ^r^epistaxis and hematemesis; ^s^overt bleeding resulting in death, decrease in Hgb by 30 g/L or more, transfusion of 2 or more units of blood, surgical intervention/decompression of closed space to stop/control the event, bleeding in retroperitoneal or intraocular location; ^t^non-major bleed; ^u^fatal bleeding, and/or bleeding in a critical area or organ, such as intracranial, intraocular, retroperitoneal, intra-articular or pericardial, or intramuscular with compartment syndrome, and/or a fall in Hgb levels of 20 g/L or greater from baseline or during any 24-hour period, and/or a transfusion of 2 U or more of PRBCs; ^v^overt non-major bleed with intervention; ^w^a fall in Hgb levels of 20 g/L or greater, and/or a transfusion of 2 U or more of PRBCs within the admission period starting from initiation of UFH order; ^x^bruising at site of injection > 5 cm diameter; ^y^hemorrhagic stroke at autopsy

### VTE outcomes

A total of 19 studies (*n* = 10079) were eligible for synthesis of the primary outcome, seven required combining PE and DVT events for assessment of VTE (Table 3). Patients had a 3.1% occurrence of VTE (12 studies, *n* = 145/4653) with TID regimens compared to 4.0% (9 studies, *n* = 218/5426) with BID regimens. A total of 21 studies (*n* = 7164) were eligible for synthesis of DVT. Patients with TID regimens experienced 4.8% DVTs (11 studies, *n* = 244/5102) compared to 9.7% with BID regimens (11 studies, *n* = 199/2062). A total of 19 studies (*n* = 7346) were eligible for synthesis of PE. Patients on TID regimens experienced fewer PEs (11 studies, 0.4%, *n* = 24/5372) compared to patients on BID regimens (9 studies, 0.9%, *n* = 17/1974).

**Table 3:** Full review analysis, general medical population subgroup analysis, and contemporary observational study subgroup analysis

Outcome	Dosing Regimen	Occurrence (%)	Quality of Studies (Good/Low Risk, Fair/Some Concerns, Poor/High Risk)	Occurrence (%)	Quality of Studies (Good/Low Risk, Fair/Some Concerns, Poor/High Risk)	Occurrence (%)	Quality of Studies (Good/Low Risk, Fair/Some Concerns, Poor/High Risk)
Key		All Studies		General Medical Population		Contemporary Observational Studies	
*Efficacy*							
VTE	BID	4.0 (9 studies, *n* = 218/5426)	(2, 2, 5)	1.6 (5 studies, *n* = 64/3961)	(2, 0, 3)	1.4 (4 studies, *n* = 54/3745)	(1, 0, 3)
	TID	3.1 (12 studies, *n* = 145/4653)	(4, 3, 5)	1.4 (9 studies, *n* = 57/4189)	(3, 3, 3)	0.8 (5 studies, *n* = 28/3467)	(3, 0, 2)
DVT	BID	9.7 (11 studies, *n* = 199/2062)	(2, 2, 7)	5.3 (4 studies, *n* = 22/419)	(1, 0, 3)	5.9 (3 studies, *n* = 12/203)	(0, 0, 3)
	TID	4.8 (11 studies, *n* = 244/5102)	(3, 3, 5)	3.6 (8 studies, *n* = 165/4638)	(2, 3, 3)	0.3 (3 studies, *n* = 8/2657)	(1, 0, 2)
PE	BID	0.9 (9 studies, *n* = 17/1974)	(2, 2, 5)	0.2 (4 studies, *n* = 1/419)	(1, 0, 3)	0.5 (3 studies, *n* = 1/203)	(0, 0, 3)
	TID	0.4 (11 studies, *n* = 24/5372)	(3, 3, 5)	0.3 (8 studies, *n* = 13/4908)	(2, 3, 3)	0.2 (3 studies, *n* = 5/2657)	(1, 0, 2)
*Safety*							
Any Bleeding	BID	3.2 (9 studies, *n* = 196/6080)	(4, 1, 4)	2.4 (5 studies, *n* = 123/5026)	(3, 0, 2)	2.5 (4 studies, *n* = 119/4803)	(2, 0, 2)
	TID	4.3 (13 studies, *n* = 393/9044)	(6, 2, 5)	4.1 (10 studies, *n* = 353/8521)	(5, 2, 3)	3.3 (6 studies, *n* = 199/6041)	(4, 0, 2)
ISTH Major Bleeding	BID	0.7 (4 studies, *n* = 27/3825)	(2, 0, 2)	0.7 (3 studies, *n* = 27/3783)	(2, 0, 1)	0.7 (2 studies, *n* = 25/3560)	(1, 0, 1)
	TID	0.6 (8 studies, *n* = 36/5777)	(3, 2, 3)	0.6 (6 studies, *n* = 31/5398)	(2, 2, 2)	0.4 (3 studies, *n* = 13/2968)	(1, 0, 2)

### Bleeding outcomes

A total of 19 studies (*n* = 15124) were eligible for synthesis of any bleeding, seven required combining different types of bleeding events for assessment of any bleeding (Table 3). Bleeding occurred in 4.3% of patients on TID regimens (13 studies, *n* = 393/9044) compared to 3.2% of patients receiving BID regimens (9 studies, *n* = 196/6080). A total of 10 studies (*n* = 9602) were eligible for synthesis of ISTH major bleeding. In patients on TID regimens, ISTH major bleeds occurred in 0.6% of patients (8 studies, *n* = 36/5777) compared to 0.7% of patients on BID regimens (4 studies, *n* = 27/3825).

### General medical population subgroup analysis

A total of 14 studies were eligible for inclusion in the general medical population subgroup analysis that excluded patients with a primary diagnosis of MI or ischemic stroke (Table 1). Three studies included a BID regimen, 8 studies included a TID regimen, and 3 studies included both BID and TID regimens. A total of 12 studies (*n* = 8150) were eligible for synthesis of the primary outcome (Table 3). Patients had a 1.4% occurrence of VTE (9 studies, *n* = 57/4189) with TID regimens compared to 1.6% (5 studies, *n* = 64/3961) with BID regimens. A total of 12 studies (*n* = 13547) were eligible for synthesis of any bleeding in the general medical population subgroup. Bleeding occurred in 2.4% of patients receiving BID regimens (5 studies, *n* = 123/5026) compared to 4.1% of patients receiving TID regimens (10 studies, *n* = 353/8521).

### Higher quality data subgroup analysis

A total of 13 studies were eligible for inclusion in the higher data quality subgroup analysis that excluded high risk of bias and poor-quality studies (Table 1). Four studies included a BID regimen, 7 studies included a TID regimen, and 2 studies included both BID and TID regimens. A total of 10 studies (*n* = 6555) were eligible for synthesis of the primary outcome (Table 4). Patients had a 3.4% occurrence of VTE (7 studies, *n* = 71/2065) with TID regimens compared to 3.9% (4 studies, *n* = 174/4490) with BID regimens. A total of 11 studies (*n* = 12300) were eligible for synthesis of any bleeding in the higher data quality subgroup. Bleeding occurred in 3.3% of patients receiving BID regimens (5 studies, *n* = 194/5878) compared to 5.2% of patients receiving TID regimens (8 studies, *n* = 332/6422).

**Table 4:** Higher data quality subgroup analysis

Outcome	Dosing Regimen	Occurrence (%)	Quality of Studies (Good/Low Risk, Fair/Some Concerns, Poor/High Risk)	Randomized Studies Only, Occurrence (%)	Observational Studies Only, Occurrence (%)
*Efficacy*					
VTE	BID	3.9 (4 studies, *n* = 174/4490)	(2, 2, 0)	14.0 (3 studies, *n* = 133/948)	1.2 (1 study, *n* = 41/3542)
	TID	3.4 (7 studies, *n* = 71/2065)	(4, 3, 0)	5.5 (4 studies, *n* = 51/920)	1.7 (3 studies, *n* = 20/1145)
DVT	BID	14.2 (4 studies, *n* = 147/1034)	(2, 2, 0)	14.2 (4 studies, *n* = 147/1034)	NA (0 studies)
	TID	7.2 (6 studies, *n* = 182/2514)	(3, 3, 0)	8.2 (5 studies, *n* = 178/2179)	1.2 (1 study, *n* = 4/335)
PE	BID	1.0 (4 studies, *n* = 10/1038)	(2, 2, 0)	1.0 (4 studies, *n* = 10/1038)	NA (0 studies)
	TID	0.4 (6 studies, *n* = 10/2784)	(3, 3, 0)	0.4 (5 studies, *n* = 9/2449)	0.3 (1 study, *n* = 1/335)
*Safety*					
Any Bleeding	BID	3.3 (5 studies, *n* = 194/5878)	(4, 1, 0)	6.4 (3 studies, *n* = 76/1185)	2.5 (2 studies, *n* = 118/4693)
	TID	5.2 (8 studies, *n* = 332/6422)	(6, 2, 0)	5.7 (4 studies, *n* = 153/2703)	4.8 (4 studies, *n* = 179/3719)
ISTH Major Bleeding	BID	0.7 (2 studies, *n* = 27/3765)	(2, 0, 0)	0.9 (1 study, *n* = 2/223)	0.7 (1 study, *n* = 25/3542)
	TID	0.8 (5 studies, *n* = 28/3349)	(3, 2, 0)	0.9 (4 studies, *n* = 23/2703)	0.8 (1 study, *n* = 5/646)

### Contemporary observational studies subgroup analysis

A total of 8 studies were eligible for inclusion in the contemporary observational studies subgroup analysis that excluded observational studies published prior to 2006 (Table 1). Two studies included a BID regimen, 3 studies included a TID regimen, and 3 studies included both BID and TID regimens. All eight studies included a general medical patient population, and no studies included patients with a primary diagnosis of MI or ischemic stroke. A total of 7 studies (*n* = 7212) were eligible for synthesis of the primary outcome (Table 3). Patients had a 0.8% occurrence of VTE (5 studies, *n* = 28/3467) with TID regimens compared to 1.4% (4 studies, *n* = 54/3745) with BID regimens. A total of 7 studies (*n* = 10844) were eligible for synthesis of any bleeding in the contemporary observational study subgroup. Bleeding occurred in 2.5% of patients receiving BID regimens (4 studies, *n* = 119/4803) compared to 3.3% of patients receiving TID regimens (6 studies, *n* = 199/6041). Additional results of all subgroup analyses are reported in Tables 3 and 4.

## Discussion

VTE prophylaxis is an important component of inpatient hospital care for most acutely ill adult patients due to the increased risk of VTE development in this population [3–5]. In our review, patients on TID prophylactic UFH regimens experienced lower occurrence of VTE, DVT, and PE, but a higher occurrence of any bleeding compared with patients on BID regimens. Occurrence of ISTH major bleeding was similar between regimens, and the largest difference in outcomes between regimens occurred with DVTs. However, the general medical population subgroup, higher data quality subgroup analyses, and contemporary observational subgroup showed similar VTE occurrence rates between regimens, warranting further examination of the data.

The most recent meta-analysis of the literature conducted by Phung et al. in 2011 compared rates of DVT, PE, major bleeding, and death in patients receiving SC low-dose UFH BID, SC low-dose UFH TID, and SC low molecular weight heparin (LMWH) as VTE prophylaxis in hospitalized, nonsurgical patients [7]. Sixteen randomized controlled trials were included in the study, five of which overlap with studies included in this review [26, 32, 33, 35, 38]. The researchers found that there was no statistically significant difference when TID UFH was compared to BID for DVT (relative risk (RR) 1.56, 95% credible interval (95% CrI) 0.64–4.33), PE (RR 1.67, 95% CrI 0.49–208.9), major bleeding (RR 0.89, 95% CrI 0.08–7.05), or death (RR 1.17, 95% CrI 0.72–1.95). Phung et al. excluded all patients presenting with acute stroke and some patients presenting with acute MI, therefore closely reflecting the general medical population subgroup analysis presented in this review. Results were similar between the general medical population subgroup analysis and Phung’s conclusions, with small differences identified between BID and TID regimens for VTE, PE, and ISTH major bleeding.

The review presented herein adds to the previous literature by including more data published recently which is reflective of current practice, though all observational (8 studies, *n* = 10937) [16–23]. Interestingly, this contemporary data shows a much lower rate of VTE overall (1.1%, 7 studies, *n* = 82/7212) and a smaller difference between dosing regimens. It is possible that with more recent practice encouraging patient mobility, rates of VTE in acutely ill hospitalized patients may be lower overall, limiting the need for greater prophylactic anticoagulation coverage with higher TID dosing regimens [39]. Additionally, in contemporary observational data VTE was generally only tested for in those with signs or symptoms of VTE as opposed to serial screening methods that were used in historical randomized, controlled trials. Asymptomatic DVTs are of uncertain clinical significance, and in current practice are not treated. Identification of these events in historical randomized, controlled trials may have overestimated the risk of morbidity in this population [40]. It is important to note that the rate of DVT and PE individually appear higher in the BID compared to TID regimen in the contemporary observational analysis, though this data may not be reliable as the BID group had a very small number (*n* = 203) and all data came from poor quality studies. Furthermore, when assessing only good and fair quality data from all observational studies included in this review, rates of VTE are much lower in both TID (1.7%, 3 studies, *n* = 20/1145) and BID (1.2%, 1 study, *n* = 41/3542) regimens than reported in the included low and some concerns of bias randomized, controlled trials for TID (5.5%, 4 studies, *n* = 51/920) and BID (14.0%, 3 studies, *n* = 133/948) regimens. With little apparent added VTE prevention with TID dosing in this contemporary good quality observational data, it would appear that twice daily dosing of prophylactic heparin is likely sufficient for most acutely ill hospitalized patients.

A preference towards BID dosing could additionally be considered due to this regimen utilizing fewer injections. A study by Popoola et al. examined the nonadherence of hospitalized patients who missed at least one dose of a VTE prophylaxis medication during their hospital stay [41]. The study concluded that SC anticoagulant doses are more likely to be missed in hospitalized patients than doses of other medication types, with patient refusal as the major driver of non-administration. Therefore, BID dosing could be preferred to improve patient adherence to SC VTE prophylactic regimens. Additional considerations related to administration of SC UFH include injection site hematoma formation and cost which may be increased with TID dosing, though not directly compared in this review.

It is important to note that not all populations of acutely ill hospitalized patients have the same risk of VTE, and this should be considered when selecting an appropriate dosing regimen. In the subgroup analysis including only studies with a general medical patient population, those with an MI or stroke were excluded. Although there is a more pronounced difference in DVT between doses in the full population, this difference is smaller in the general medical population subgroup. This could suggest that the increased baseline risk of VTE in those with an MI or stroke may warrant TID UFH. Additionally, the smaller difference between DVT occurrence in the general medical population subgroup, as well as smaller differences in the occurrence of VTE and PE, may suggest a preference towards BID UFH for the general medical population.

Our study has various limitations that should be considered while interpreting results. First, not all data were consistently reported in each study, so only studies that included the outcome could be assessed for each outcome. Additionally, the more robust type of data (per-protocol for effectiveness, total population for safety) was utilized when available. The investigators did synthesize the occurrence of the primary outcome of VTE by adding the individual occurrence of DVT and PE for studies that did not report a total VTE event rate. Although DVT and PE events were only added if they were reported from the same patient population, this introduces a risk of overestimating the rate of VTE occurrence in this review if one patient experienced multiple VTE events. This same approach was used when evaluating the secondary outcome of any bleeding in which researchers added the events of individual types of bleeds for studies that did not report a total number of bleeds. Although types of bleeding were only added if they occurred in the same patient population, the same risk of overestimating bleeding rate is present. Bleeding definitions were not consistent across trials making events difficult to assess, though an analysis grouping bleeds into ISTH major bleeding was conducted where possible. Another limitation to this review is the wide range of data quality and publication dates of the included studies. Due to injectable UFH achieving FDA approval in 1939, this review includes many studies that were published in the 1980s and 1990s that may not be reflective of current practice [6]. As a result, included studies and our synthesized outcomes include a mix of data quality as well as changes in standards of care. To address this limitation, individual study quality of each synthesized outcome was reported and a subgroup analysis assessing only higher quality data was conducted. Furthermore, few studies included in this review reported the race or ethnicity of their patient population. Therefore, although this review applies to acutely ill hospitalized adults, it is unclear if there is a specific race or ethnicity that this review more directly applies to. Additionally, due to the varied data provided in each study included in the review, confounders were not able to be controlled for throughout the studies. Where available, patient demographics and disease severity were provided but unable to be accounted for in the analysis. A subgroup of general medical patients was conducted to eliminate any admitting diagnoses that may lead to a higher thrombotic rate such as myocardial infarction or stroke, but severity of illness was not able to be further assessed.

## Conclusion

There is an increased risk of VTE for most acutely ill hospitalized adults. This increased risk often warrants pharmacologic VTE prophylaxis with medications such as SC UFH. This review found that patients on TID 5000U SC UFH regimens experienced fewer occurrences of VTE, DVT, and PE, but more occurrence of any bleeding compared to patients on BID regimens. However, newer good quality observational data shows a more similar occurrence of VTE between regimens. Therefore, in the majority of acutely ill hospitalized adults, BID regimens may be appropriate to balance thrombotic and bleeding risk. In patients admitted to medical floors with acute myocardial infarction or stroke, the thrombotic burden may warrant TID dosing for greater thromboprophylaxis.

## Data Availability

The following statements are declared in the manuscript: “…registered with PROSPERO CRD42023493327 “Full search strategies are shared via Open Science Framework.” (OSF link: https://osf.io/k5va4/?view_only=e44e7458dd4e417392cd4f114f30a2b1).

## References

[1] Heit JA, O’Fallon WM, Petterson TM et al (2002) Relative impact of risk factors for deep vein thrombosis and pulmonary embolism: a population-based study. Arch Intern Med Jun 10(11):1245–1248. 10.1001/archinte.162.11.1245 10.1001/archinte.162.11.1245 12038942

[2] Grosse SD, Nelson RE, Nyarko KA, Richardson LC, Raskob GE (2016) The economic burden of incident venous thromboembolism in the united states: A review of estimated attributable healthcare costs. Thromb Res Jan 137:3–10. 10.1016/j.thromres.2015.11.033 10.1016/j.thromres.2015.11.033 PMC4706477 26654719

[3] Barbar S, Noventa F, Rossetto V et al (2010) A risk assessment model for the identification of hospitalized medical patients at risk for venous thromboembolism: the Padua prediction score. J Thromb Haemost Nov 8(11):2450–2457. 10.1111/j.1538-7836.2010.04044.x 10.1111/j.1538-7836.2010.04044.x 20738765

[4] Schünemann HJ, Cushman M, Burnett AE et al (2018) American society of hematology 2018 guidelines for management of venous thromboembolism: prophylaxis for hospitalized and nonhospitalized medical patients. Blood Adv Nov 27(22):3198–3225. 10.1182/bloodadvances.2018022954 10.1182/bloodadvances.2018022954 PMC6258910 30482763

[5] Kahn SR, Lim W, Dunn AS et al (2012) Prevention of VTE in nonsurgical patients: antithrombotic therapy and prevention of thrombosis, 9th ed: American college of chest physicians Evidence-Based clinical practice guidelines. Chest Feb 141(2 Suppl):e195S–e226. 10.1378/chest.11-2296 10.1378/chest.11-2296 PMC3278052 22315261

[6] Heparin Sodium Injection [package insert] (2024) Eatontown, NJ: Hikma Pharmaceuticals USA Inc. https://www.accessdata.fda.gov/drugsatfda_docs/label/2024/017037s201lbl.pdf. Published October 24, Accessed November 1, 2024

[7] Phung OJ, Kahn SR, Cook DJ, Murad MH (2011) Dosing frequency of unfractionated heparin thromboprophylaxis: a meta-analysis. Chest Aug 140(2):374–381. 10.1378/chest.10-3084 10.1378/chest.10-3084 21349929

[8] Page MJ, McKenzie JE, Bossuyt PM et al (2021) The PRISMA 2020 statement: an updated guideline for reporting systematic reviews. Bmj Mar 29:372:n71. 10.1136/bmj.n71 10.1136/bmj.n71 PMC8005924 33782057

[9] Lefebvre C, Glanville J, Briscoe S et al (2023) Chapter 4: Searching for and selecting studies. In: Higgins JPT, Thomas J, Chandler J, eds. *Cochrane handbook for systematic reviews of interventions.* Cochrane; Accessed November 1, 2023. https://training.cochrane.org/handbook/current/chapter-04

[10] Schulman S, Kearon C (2005) Definition of major bleeding in clinical investigations of antihemostatic medicinal products in non-surgical patients. J Thromb Haemost Apr 3(4):692–694. 10.1111/j.1538-7836.2005.01204.x 10.1111/j.1538-7836.2005.01204.x 15842354

[11] Covidence systematic review software. Veritas health innovation. www.covidence.org

[12] Wells GA, Shea B, O’Connell D, Peterson J, Welch V, Losos M The Newcastle-Ottawa Scale (NOS) for assessing the quality if nonrandomized studies in meta-analyses. https://www.ohri.ca/programs/clinical_epidemiology/oxford.asp

[13] Sterne JAC, Savović J, Page MJ et al (2019) RoB 2: a revised tool for assessing risk of bias in randomised trials. Bmj Aug 28:366:l4898. 10.1136/bmj.l4898 10.1136/bmj.l4898 31462531

[14] Forbes C, Greenwood H, Carter M, Clark J (2024) Automation of duplicate record detection for systematic reviews: deduplicator. Syst Rev Aug 2(1):206. 10.1186/s13643-024-02619-9 10.1186/s13643-024-02619-9 PMC11295717 39095913

[15] Kamran SI, Downey D, Ruff RL (1998) Pneumatic sequential compression reduces the risk of deep vein thrombosis in stroke patients. Neurology 50(6):1683–1688. 10.1212/wnl.50.6.1683 9633711 10.1212/wnl.50.6.1683

[16] Peterman CM, Kolansky DM, Spinler SA (2006) Prophylaxis against venous thromboembolism in acutely ill medical patients: an observational study. Pharmacotherapy 26(8):1086–1090. 10.1592/phco.26.8.1086 16863485 10.1592/phco.26.8.1086

[17] Ishida T, Suzuki T, Watanabe K, Sakurai H, Uchida H, Mimura M (2014) Prophylactic use of heparin for deep vein thrombosis in restrained psychiatric patients: a chart review. Gen Hosp Psychiatry 36(6):690–693. 10.1016/j.genhosppsych.2014.06.007 25070076 10.1016/j.genhosppsych.2014.06.007

[18] Beall J, Woodruff A, Hempel C, Wovkulich M, Zammit K (2016) Efficacy and safety of High-Dose subcutaneous unfractionated heparin prophylaxis for the prevention of venous thromboembolism in obese hospitalized patients. Hosp Pharm 51(5):376–381. 10.1310/hpj5105-376 27303091 10.1310/hpj5105-376 PMC4896346

[19] Joy M, Tharp E, Hartman H et al (2016) Safety and efficacy of High-Dose unfractionated heparin for prevention of venous thromboembolism in overweight and obese patients. Pharmacotherapy 36(7):740–748. 10.1002/phar.1775 27265806 10.1002/phar.1775

[20] Regis T, Goriacko P, Ferguson N (2021) Safety of High-Dose unfractionated heparin for prophylaxis of venous thromboembolism in hospitalized obese patients. Ann Pharmacother 55(8):963–969. 10.1177/1060028020974569 33215504 10.1177/1060028020974569

[21] Sorgi MW, Roach E, Bauer SR et al (2022) Effectiveness and safety of twice daily versus thrice daily subcutaneous unfractionated heparin for venous thromboembolism prophylaxis at a tertiary medical center. J Pharm Pract 35(2):190–196. 10.1177/0897190020961210 33016183 10.1177/0897190020961210

[22] Jatis AJ, Nei SD, Zieminski JJ, Mara K, Krauter AK (2023) Assessment of bleeding risk in low-weight patients receiving prophylactic subcutaneous unfractionated heparin. Vasc Med 28(5):443–448. 10.1177/1358863x231189758 37555546 10.1177/1358863X231189758

[23] Kaylor DM, Wade RM, Chappell KB, Niemann MH, VanArsdale VM (2023) Venous thromboembolism prophylaxis with Enoxaparin versus unfractionated heparin in patients with low body weight. Plasmatology 17. 10.1177/26348535231156848

[24] Warlow C, Beattle AG, Terry G (1973) A double blind trial of low doses of subcutaneous heparin in the prevention of deep vein thrombosis after myocardial infarction. Lancet 2(7835):934–936 4126560 10.1016/s0140-6736(73)92597-x

[25] Gelmers HJ (1980) Effects of low-dose subcutaneous heparin on the occurrence of deep vein thrombosis in patients with ischemic stroke. Acta Neurol Scand 61(5):313–318. 10.1111/j.1600-0404.1980.tb01498.x 7386175 10.1111/j.1600-0404.1980.tb01498.x

[26] Belch JJ, Lowe GD, Ward AG, Forbes CD, Prentice CR (1981) Prevention of deep vein thrombosis in medical patients by low-dose heparin. Scott Med J 26(2):115–117. 10.1177/003693308102600205 7291971 10.1177/003693308102600205

[27] McCarthy ST, Turner J (1986) Low-dose subcutaneous heparin in the prevention of deep-vein thrombosis and pulmonary emboli following acute stroke. Age Ageing 15(2):84–88. 10.1093/ageing/15.2.84 3962762 10.1093/ageing/15.2.84

[28] Zawilska K, Psuja P, Lewandowski K, Wróz M (1989) Low-dose heparin in the prevention of thrombotic complications following acute myocardial infarction. Cor Vasa 31(3):179–185 2670447

[29] Harenberg J, Kallenbach B, Martin U et al (1990) Randomized controlled study of heparin and low molecular weight heparin for prevention of deep-vein thrombosis in medical patients. Thromb Res 59(3):639–650. 10.1016/0049-3848(90)90422-9 2173168 10.1016/0049-3848(90)90422-9

[30] Turpie AG, Gent M, Côte R et al (1992) A low-molecular-weight heparinoid compared with unfractionated heparin in the prevention of deep vein thrombosis in patients with acute ischemic stroke. A randomized, double-blind study. Ann Intern Med 117(5):353–357. 10.7326/0003-4819-117-5-353 1503326 10.7326/0003-4819-117-5-353

[31] Dumas R, Woitinas F, Kutnowski M et al (1994) A multicentre, double-blind, randomized study to compare the safety and efficacy of once-daily ORG 10172 and twice-daily low-dose heparin in preventing deep-vein thrombosis in patients with acute ischaemic stroke. Age Ageing 23(6):512–516. 10.1093/ageing/23.6.512 9231947 10.1093/ageing/23.6.512

[32] Bergmann JF, Neuhart E (1996) A multicenter randomized double-blind study of Enoxaparin compared with unfractionated heparin in the prevention of venous thromboembolic disease in elderly in-patients bedridden for an acute medical illness. Thromb Haemost 76(4):529–534. 10.1055/s-0038-1650617 8902991

[33] Lechler E, Schramm W, Flosbach CW (1996) The venous thrombotic risk in non-surgical patients: epidemiological data and efficacy/safety profile of a low-molecular-weight heparin (enoxaparin). The prime study group. Haemostasis 26(Suppl 2):49–56. 10.1159/000217272 8707167 10.1159/000217272

[34] Hillbom M, Erilä T, Sotaniemi K, Tatlisumak T, Sarna S, Kaste M (2002) Enoxaparin vs heparin for prevention of deep-vein thrombosis in acute ischaemic stroke: a randomized, double-blind study. Acta Neurol Scand 106(2):84–92. 10.1034/j.1600-0404.2002.01215.x 12100367 10.1034/j.1600-0404.2002.01215.x

[35] Kleber FX, Witt C, Vogel G, Koppenhagen K, Schomaker U, Flosbach CW (2003) Randomized comparison of Enoxaparin with unfractionated heparin for the prevention of venous thromboembolism in medical patients with heart failure or severe respiratory disease. Am Heart J 145(4):614–621. 10.1067/mhj.2003.189 12679756 10.1067/mhj.2003.189

[36] Diener H, Ringelstein EB, von Kummer R et al (2006) Prophylaxis of thrombotic and embolic events in acute ischemic stroke with the low-molecular-weight heparin certoparin: results of the PROTECT trial. Stroke (00392499) 37(1):139–144. 10.1161/01.str.0000195182.67656.ee 10.1161/01.STR.0000195182.67656.ee 16306456

[37] Sherman DG, Albers GW, Bladin C et al (2007) The efficacy and safety of Enoxaparin versus unfractionated heparin for the prevention of venous thromboembolism after acute ischaemic stroke (PREVAIL Study): an open-label randomised comparison. Lancet 369(9570):1347–1355. 10.1016/s0140-6736(07)60633-3 17448820 10.1016/S0140-6736(07)60633-3

[38] Riess H, Haas S, Tebbe U et al (2010) A randomized, double-blind study of certoparin vs. unfractionated heparin to prevent venous thromboembolic events in acutely ill, non-surgical patients: CERTIFY study. J Thromb Haemost 8(6):1209–1215. 10.1111/j.1538-7836.2010.03848.x 20218984 10.1111/j.1538-7836.2010.03848.x

[39] Neeman E, Liu V, Mishra P et al (2022) Trends and risk factors for venous thromboembolism among hospitalized medical patients. JAMA Netw Open Nov 1(11):e2240373. 10.1001/jamanetworkopen.2022.40373 10.1001/jamanetworkopen.2022.40373 PMC9679881 36409498

[40] Yamashita Y, Shiomi H, Morimoto T et al (2017) Asymptomatic lower extremity deep vein Thrombosis - Clinical characteristics, management strategies, and Long-Term outcomes. Circ J Nov 24(12):1936–1944. 10.1253/circj.CJ-17-0445 10.1253/circj.CJ-17-0445 28659542

[41] Popoola VO, Lau BD, Tan E et al (2018) Nonadministration of medication doses for venous thromboembolism prophylaxis in a cohort of hospitalized patients. Am J Health Syst Pharm Mar 15(6):392–397. 10.2146/ajhp161057 10.2146/ajhp161057 29523536

